# Proteomic profile of the *Bradysia odoriphaga* in response to the microbial secondary metabolite benzothiazole

**DOI:** 10.1038/srep37730

**Published:** 2016-11-24

**Authors:** Yunhe Zhao, Kaidi Cui, Chunmei Xu, Qiuhong Wang, Yao Wang, Zhengqun Zhang, Feng Liu, Wei Mu

**Affiliations:** 1 College of Plant Protection, Shandong Provincial Key Laboratory for Biology of Vegetable Diseases and Insect Pests, Shandong Agricultural University, Tai’an, Shandong 271018, P.R. China; 2 College of Horticultural Science and Engineering, Shandong Agricultural University, Tai’an, Shandong 271018, PR China

## Abstract

Benzothiazole, a microbial secondary metabolite, has been demonstrated to possess fumigant activity against *Sclerotinia sclerotiorum*, *Ditylenchus destructor* and *Bradysia odoriphaga*. However, to facilitate the development of novel microbial pesticides, the mode of action of benzothiazole needs to be elucidated. Here, we employed iTRAQ-based quantitative proteomics analysis to investigate the effects of benzothiazole on the proteomic expression of *B. odoriphaga*. In response to benzothiazole, 92 of 863 identified proteins in *B. odoriphaga* exhibited altered levels of expression, among which 14 proteins were related to the action mechanism of benzothiazole, 11 proteins were involved in stress responses, and 67 proteins were associated with the adaptation of *B. odoriphaga* to benzothiazole. Further bioinformatics analysis indicated that the reduction in energy metabolism, inhibition of the detoxification process and interference with DNA and RNA synthesis were potentially associated with the mode of action of benzothiazole. The myosin heavy chain, succinyl-CoA synthetase and Ca^+^-transporting ATPase proteins may be related to the stress response. Increased expression of proteins involved in carbohydrate metabolism, energy production and conversion pathways was responsible for the adaptive response of *B. odoriphaga*. The results of this study provide novel insight into the molecular mechanisms of benzothiazole at a large-scale translation level and will facilitate the elucidation of the mechanism of action of benzothiazole.

Chinese chive (*Allium tuberosum* Rottler) is a hardy perennial herbaceous vegetable of high economic value in several regions of southeastern and eastern Asia^1^,^2^. A major factor restricting Chinese chive production is the chive maggot, *Bradysia odoriphaga* (Diptera: Sciaridae)^3^,^4^. The larvae feed on the roots and bulbs of chives, making it difficult to control them using common strategies, and cause more than 50% of production losses in the absence of insecticidal protection^3^. One of the most prevalent management practices for controlling *B. odoriphaga* is the application of synthetic insecticides (such as organophosphates, carbamates, and neonicotinoids) in China and elsewhere^5^. However, the control efficacy is unsatisfactory because of the dilution effects of soil and water on pesticides and the overlapping generations of *B. odoriphaga*^6^. This challenge has led to the excessive application of chemical insecticides, which has resulted in the development of insecticide resistance in *B. odoriphaga* and high residue levels being left on marketed Chinese chives^4^. Reducing the application of these insecticides will require the development of novel effective insecticides as an alternative to conventional ones.

Benzothiazole is one component of the volatile organic compounds (VOCs) derived from microbial secondary metabolites^7^,^8^. This compound has been demonstrated to be a fumigant that can be used in the control of *Sclerotinia sclerotiorum*^9^, *Ditylenchus destructor*^10^, and *Tribolium castaneum*^11^. In our previous study, we found that benzothiaz ole exhibited fumigation toxicity to all stages of *B. odoriphaga*^12^, decreased the fecundity of female adults, and prolonged the developmental time of *B. odoriphaga*^13^, thus indicating its potential as a fumigant for the control of this pest. Moreover, the respiratory rate of *B. odoriphaga* was significantly increased in the beginning of benzothiazole treatment, and as the fumigation time was extended, the respiratory rate was significantly reduced^12^. These results indicate that benzothiazole is not a respiratory inhibitor. In addition, benzothiazole can cause a significant reduction in food consumption and can decrease nutrient accumulation in *B. odoriphaga* larvae by disrupting the activity of digestive enzymes^14^. Most studies of benzothiazole as a broad-spectrum fumigant against pests have focused on the determination of its biological activity. However, the molecular mechanism of action of benzothiazole remains poorly understood, and understanding this mechanism will be helpful for the development of new pesticides and the control of these pests in the future.

In recent years, proteomics analysis has emerged as a powerful method for studying changes in protein expression profiles at the cellular level in response to various stresses^15^,^16^,^17^,^18^,^19^. This approach has been widely used to identify the modes of action of some drugs and in target discovery^20^,^21^,^22^. A wide range of studies has been conducted utilizing iTRAQ-based quantitative proteomic technology^23^,^24^,^25^,^26^,^27^ because of its high proteome coverage, high sensitivity and labeling efficiency. For instance, Pang *et al*. demonstrated that the mechanism of action of pyrimorph in *Phytophthora capsici* involves the inhibition of cell wall biosynthesis using iTRAQ-based quantitative proteomics^24^. Thus, the use of proteomics approaches is useful for elucidating the mode of action of novel pesticides.

In the present study, we utilized an iTRAQ-based quantitative proteomic approach to analyze proteomic changes in *B. odoriphaga* in response to benzothiazole. Our goals were to identify proteins that are differentially expressed in *B. odoriphaga* following treatment with benzothiazole. An analysis of these proteins provides important insights into the mechanism of action of benzothiazole.

## Results

### Motility and ingestion of *B. odoriphaga*

**Figure 1:**
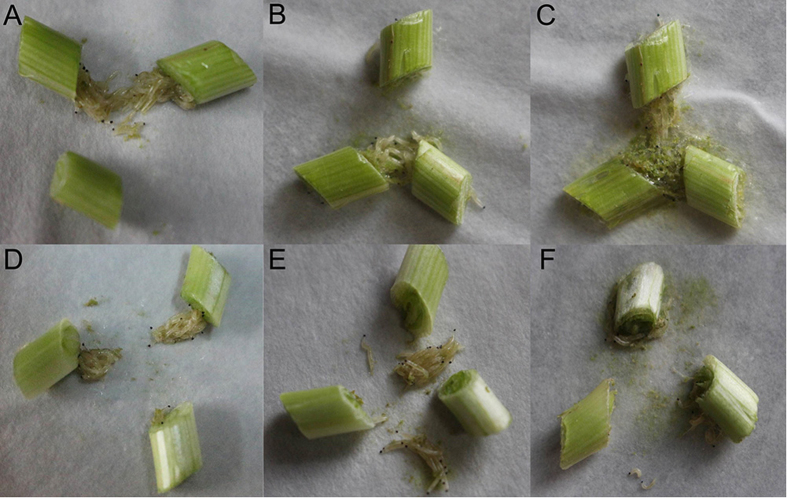
The motility and ingestion of *B. odoriphaga* after treatment with distilled water for 0 h (**A**), 6 h (**B**) and 24 h (**C**) and with the LC_30_ of benzothiazole for 0 h (**D**), 6 h (**E**) and 24 h (**F**).

As shown in Fig. 1, the effects of benzothiazole on the motility and ingestion of *B. odoriphaga* were observed at 0 h, 6 h and 24 h after treatment. At the beginning of fumigation (0 h), larvae gathered near the Chinese chive rhizomes and ingested the rhizomes both in the distilled water (Fig. 1A) and the benzothiazole (Fig. 1D) treatments. Following distilled water and benzothiazole treatment for 6 h, the larvae drilled into and ingested the fresh Chinese chive rhizomes in the distilled water treatment (Fig. 1B). However, in the benzothiazole treatment, the larvae gathered around the rhizomes but exhibited almost no ingestion or activity (Fig. 1E). Following distilled water treatment for 24 h, the larvae drilled into and ingested the rhizomes, surrounded by the secretion of silk thread and food debris (Fig. 1C). After benzothiazole treatment for 24 h, the surviving larvae had adapted and recovered their ingestion and movement (Fig. 1F).

### Overview of the quantitative proteomics analysis

**Figure 2:**
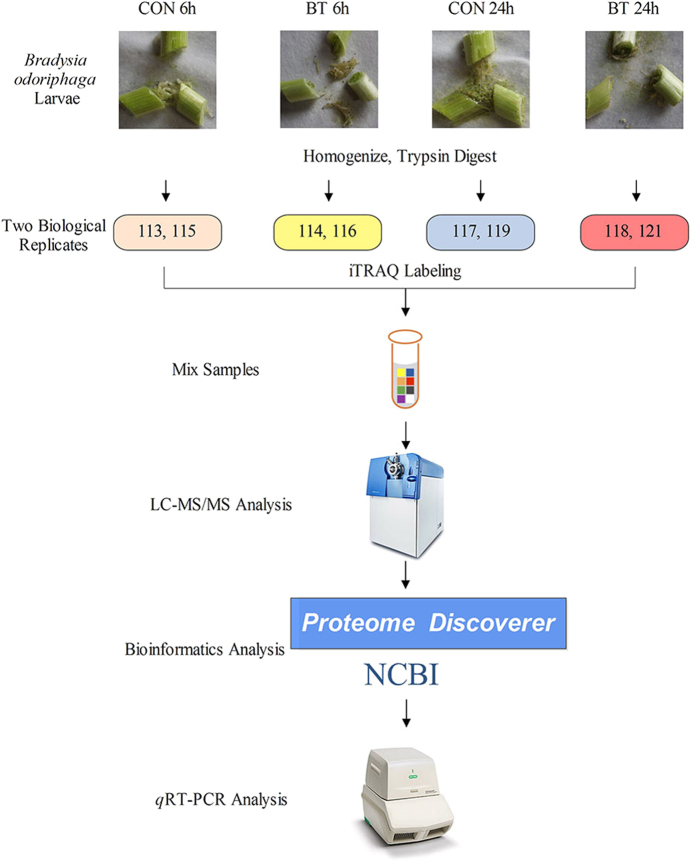
Experimental design and schematic diagram of the workflow of this study. CON: control, BT: benzothiazole.

**Figure 3:**
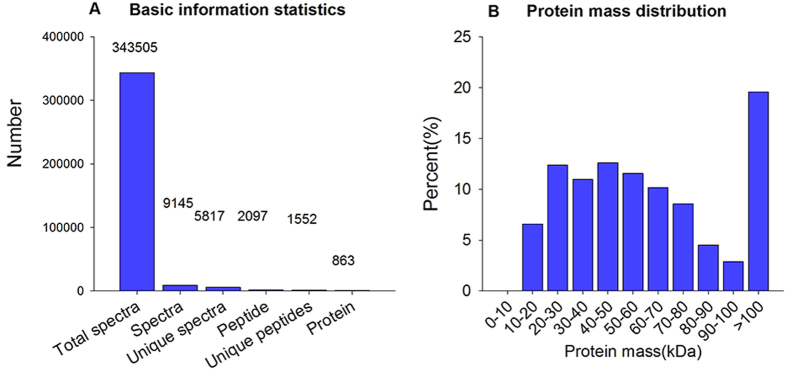
(**A**) Spectra, peptides and proteins identified from iTRAQ proteomics by searching against the Nematocera database. (**B**) Molecular weight distribution of the proteins that were identified from the iTRAQ analysis of *B. odoriphaga*.

Figure 2 shows the workflow of iTRAQ-based quantitative proteomic analysis and some proteins that were verified by *q*RT-PCR in this study. A total of 863 proteins were identified on the basis of 9,145 highly confident spectra, of which 1,552 peptides were unique (Fig. 3A). In terms of protein mass distribution, good coverage was obtained for a wide range for proteins larger than 10 kDa (Fig. 3B).

**Figure 4:**
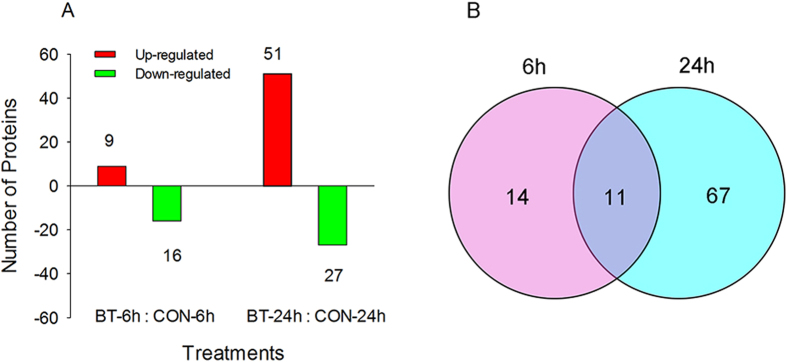
(**A**) The number of up- and down-regulated proteins of *B. odoriphaga* after treatment with benzothiazole for 6 and 24 h. BT: benzothiazole; CON: control. (**B**) Venn diagram showing the overlap between the differentially expressed proteins of *B. odoriphaga* at 6 and 24 h after benzothiazole treatment.

Using a threshold of a 1.2-fold change in abundance (±) and a *p*-value less than 0.05 (compared to the distilled water-treated larvae), 92 unique proteins were found to have significantly changed in abundance when *B. odoriphaga* larvae were treated with benzothiazole for 6 h and 24 h (Tables 1, 2, 3, [Table t1], [Table t1], [Table t2], [Table t3]). Of these unique proteins, the abundance of 25 (9 up-regulated and 16 down-regulated) was changed significantly after 6 h, and the abundance of 78 (51 up-regulated and 27 down-regulated) was changed significantly after 24 h (Fig. 4A). Among these proteins with altered abundance, 11 were shared between 6 h and 24 h, whereas the more responsive proteins were unique to the different treatment times (Fig. 4B).

### Proteins related to the mode of action of benzothiazole

**Figure 5:**
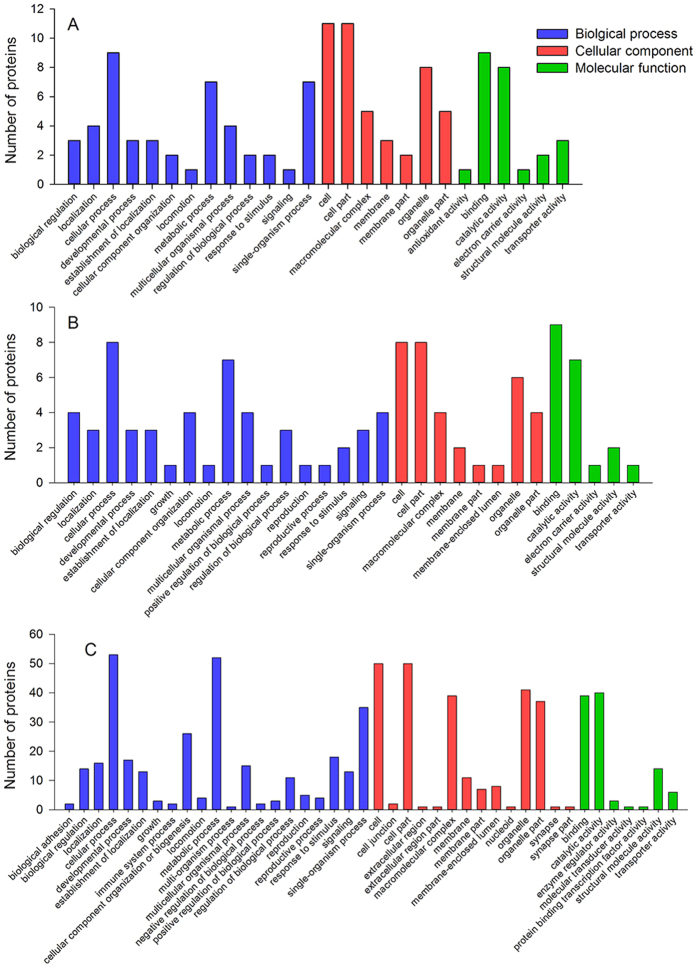
Gene ontology (GO) enrichment analysis of the differentially expressed proteins. The proteins are grouped into three GO terms: biological process, cellular component, and molecular function. (**A**) Proteins related to the action mechanism; (**B**) proteins related to the stress response; (**C**) proteins related to the adaption response.

Proteins that were differently expressed under benzothiazole treatment at 6 h but not at 24 h were considered to be related to the mode of action of benzothiazole on *B. odoriphaga*. In total, 14 proteins were differentially expressed, of which 2 were up-regulated and 12 were down-regulated (Table 1). GO enrichment analysis was used to categorize these proteins into biological processes, cellular components and molecular functions. The results are presented in Fig. 5A. The categories affected by benzothiazole mainly involved metabolic process, cellular process, single-organism process, binding, catalytic activity, and transporter activity. A COG analysis classified these 14 proteins into 10 functional groups, including a range of metabolic pathways, such as carbohydrate metabolism, lipid metabolism, nucleotide metabolism and inorganic ion metabolism, and cytoskeleton, energy production and conversion, and general function predictions (Table 1).

### Proteins related to the stress response of *B. odoriphaga* to benzothiazole

The *B. odoriphaga* proteins that were significantly changed by the benzothiazole treatment at 6 and 24 h were related to the stress response. Eleven differentially expressed proteins were identified, of which 6 were up-regulated, 2 were down-regulated and 3 were up- or down-regulated at different times (Table 2). GO analysis was conducted to categorize these proteins, and the most-assigned classifications included metabolic process, cellular process, single-organism process, binding, catalytic activity, and structural molecule activity (Fig. 5B). COG classification showed that most proteins were involved in “posttranslational modification, protein turnover, chaperones”, “translation, ribosomal structure and biogenesis”, energy production and conversion, and cytoskeleton (Table 2).

### Proteins related to the adaptation response of *B. odoriphaga* to benzothiazole

Proteins that were significantly affected by the benzothiazole treatment at 24 h but not at 6 h were considered to be related to the adaptation response of *B. odoriphaga* to benzothiazole. A total of 67 significantly affected proteins (43 up-regulated and 24 down-regulated) were identified (Table 3). These proteins were assigned to GO categories. The main enrichment categories were cellular and metabolic processes, cell and cell part, organelle, binding, and catalytic activity (Fig. 5C). The COG analysis categorized these 67 proteins into 16 functional groups, and the most frequently detected functional categories were amino acid transport and metabolism, carbohydrate transport and metabolism, energy production and conversion, “posttranslational modification, protein turnover, chaperones”, “translation, ribosomal structure and biogenesis”, general function prediction and signal transduction mechanisms (Table 3).

### *q*RT-PCR analysis of differentially expressed proteins

**Figure 6:**
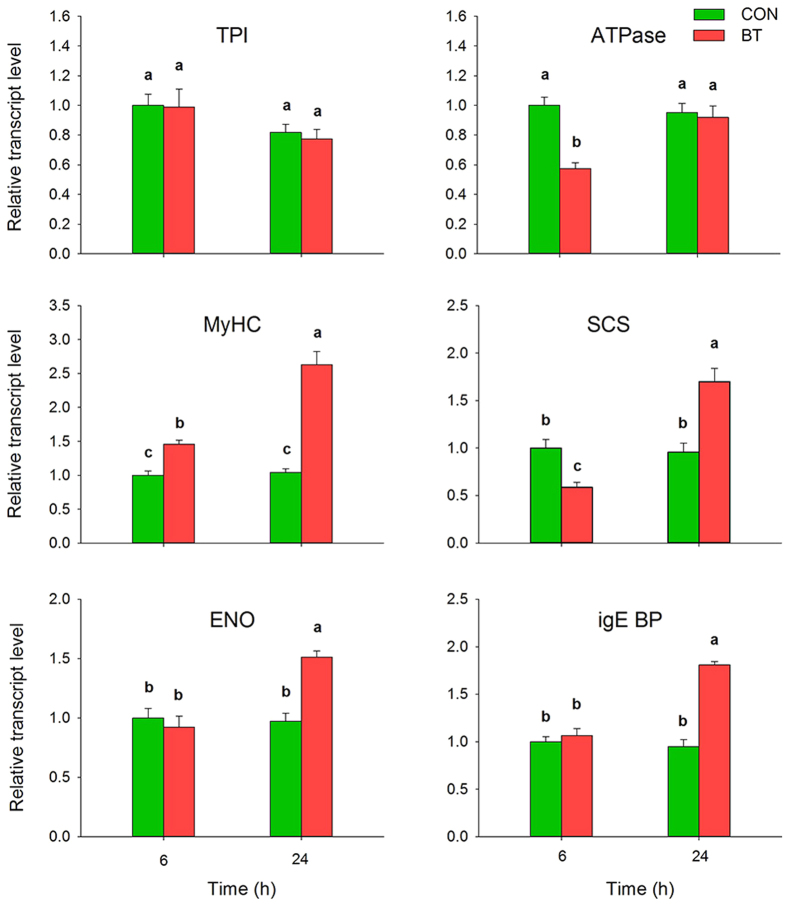
mRNA expression level analysis (qRT-PCR) of 6 proteins of *B. odoriphaga* after 6 and 24 h of benzothiazole treatment. The relative expression level was normalized to an internal standard, ribosomal protein S3 (RPS3). Bars represent mean ± SE (n = 6). Different lower-case letters above the bars indicate significant differences at *P* < 0.05. TPI: triose-phosphate isomerase; V-ATPase: vacuolar ATP synthase subunit H; MyHC: myosin heavy chain; SCS: succinyl-CoA synthetase; ENO: enolase; epsilon BP: IgE binding protein.

To determine whether gene expression is correlated between mRNA and protein levels, the following six proteins that are mainly involved in the categories of action mechanism, stress mechanism and adaption mechanism were selected for *q*RT-PCR analysis: triose-phosphate isomerase (TPI), vacuolar ATP synthase subunit H (V-ATPase), myosin heavy chain (MyHC), succinyl-CoA synthetase alpha (SCS), putative enolase (ENO) and putative IgE binding protein (epsilon BP). The expression levels of all selected genes encoding these proteins, with the exception of TPI, matched well with the iTRAQ results (Fig. 6). According to the *q*RT-PCR results, these genes had similar mRNA and protein expression patterns with similar or slightly different overall quantitative proteomics results.

## Discussion

In this study, an iTRAQ-based proteomics approach was used to quantitatively describe changes in the protein profile of *B. odoriphaga* that occur in response to the microbial secondary metabolite benzothiazole. The iTRAQ-coupled LC-MS/MS analysis identified 863 proteins, of which 92 showed altered expression. Motility observations showed that *B. odoriphaga* larvae were poisoned after 6 h of benzothiazole and recovered after treatment for 24 h. To understand how protein expression responded to benzothiazole, the differentially expressed proteins were divided into three categories: those related to the action mechanism, to the stress mechanism and to the adaption mechanism.

Among the 14 proteins that exhibited altered expression upon benzothiazole treatment, most were involved in energy production and carbohydrate and nucleotide metabolism, including triose-phosphate isomerase (TPI), vacuolar ATP synthase subunit H (V-ATPase), putative peroxisomal 3-ketoacyl-CoA thiolase (KAT), superoxide dismutase (SOD), dihydropyrimidine dehydrogenase (DPD), and nucleoside diphosphate kinase (NDPK).

**Figure 7:**
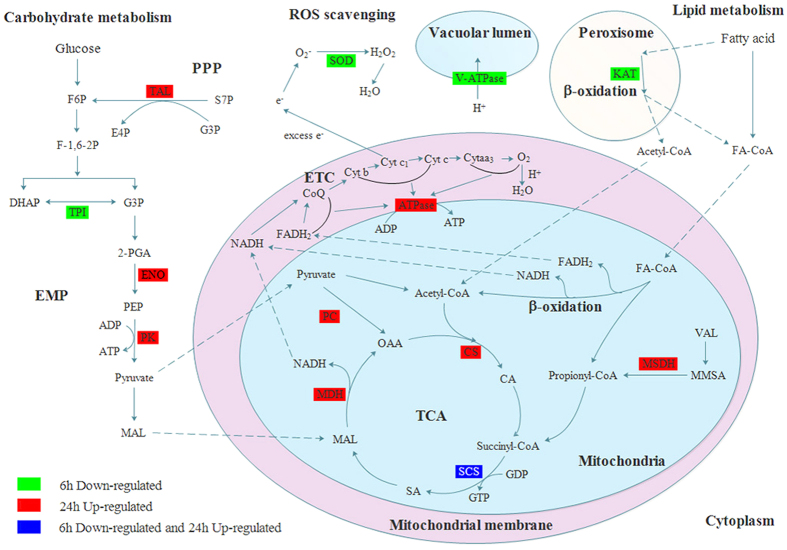
A summary of some of the biological pathways affected by benzothiazole in *B. odoriphaga*. Green boxes represent proteins that were only down-regulated at 6 h after benzothiazole treatment, red boxes represent proteins that were only up-regulated at 24 h after benzothiazole treatment, and blue boxes indicate proteins that were down-regulated at 6 h and up-regulated at 24 h after benzothiazole treatment. EMP: glycolytic pathway; PPP: pentose phosphate pathway; TCA: tricarboxylic acid cycle; ETC: electron transfer chain; F6P: fructose 6-phosphate; F-1,6-2 P: fructose-1,6-diphosphate; TAL: putative transaldolase; S7P: sedoheptulose-7-phosphate; G3P: glyceraldehyde 3-phosphate; E4P: erythrose-4-phosphate; TPI: triose-phosphate isomerase; DHAP: dihydroxyacetone phosphate; 2-PGA: 2-phosphoglycerate; ENO: putative enolase; PEP: phosphoenolpyruvate; PK: pyruvate kinase; MAL: malate; PC: pyruvate carboxylase; OAA: oxaloacetic acid; CS: citrate synthase 1; CA: citric acid; SCS: succinyl-CoA synthetase alpha; SA: succinic acid; MDH: malate dehydrogenase; VAL: valine; MMSA: methylmalonate semialdehyde; MSDH: methylmalonate semialdehyde dehydrogenase; FA-CoA: acyl-coenzyme A; KAT: peroxisomal 3-ketoacyl-CoA thiolase; ATPase: ATP synthase beta subunit; V-ATPase: vacuolar ATP synthase subunit H; SOD: superoxide dismutase.

Triose-phosphate isomerase (TPI) is a key enzyme of glycolysis and gluconeogenesis and plays a vital role in the development and metabolism of organisms^28^. TPI catalyzes the interconversion of dihydroxyacetone phosphate (DHAP) and glyceraldehyde 3-phosphate (G3P) (Fig. 7). G3P can be further processed to pyruvate, permitting the generation of NADH and ATP. Thus, TPI enables these three-carbon compounds to be processed via glycolytic metabolism. No ATP would be produced in the glycolytic pathway without this reaction^29^. The reduced level of TPI detected here indicates that benzothiazole inhibits energy production by affecting glycolytic processes.

In addition, vacuolar ATP synthase subunit H (V-ATPase) is a universal and vital component of eukaryotic organisms because it is the major proton pump of vacuolar membranes (Fig. 7). V-ATPase is a multi-subunit enzyme that comprises a membrane sector and a cytosolic catalytic sector and plays a major role in providing energy for several secondary uptake cellular processes^30^. The decreased expression of vacuolar ATP synthase subunit H suggests that benzothiazole inhibits the energy production and cellular uptake processes of *B. odoriphaga*. This provides further evidence that benzothiazole treatment can inhibit the energy production of *B. odoriphaga*. This is consistent with our observations that the larvae were barely able to crawl or ingest at 6 h after benzothiazole treatment (Fig. 2E).

The putative enzyme peroxisomal 3-ketoacyl-CoA thiolase (KAT) catalyzes the final step of fatty acid β-oxidation in the peroxisome (Fig. 7), which involves the thiolytic cleavage of 3-ketoacyl-CoA to acetyl-CoA (C_2_) and acyl-CoA (C_n−2_)^31^,^32^. Acetyl-CoA is a central molecule derived from glucose, fatty acid, and amino acid catabolism and is involved in many metabolic pathways and transformations^33^. In addition, acetyl-CoA is preferentially directed into the mitochondria for the synthesis of ketone bodies and ATP^34^. Down-regulation of peroxisomal 3-ketoacyl-CoA thiolase inhibits the generation of acetyl-CoA and decreases the synthesis of ATP. The down-regulation of this set of proteins suggests that the mechanism of action of benzothiazole is related to the inhibition of energy production in *B. odoriphaga*.

Dihydropyrimidine dehydrogenase (DPD) is the initial and rate-limiting enzyme in the pyrimidine catabolic pathway, in which thymine and uracil are converted into β-alanine or β-aminoisobutyrate using the cofactors NADH or NADPH^35^,^36^. β-alanine is involved in many metabolic pathways and neurotransmitter functions^37^. DPD down-regulation leads to the disruption of normal pyrimidine metabolism. Additionally, DPD is a crucial enzyme for the growth and survival of the parasite under a glucose-limited environment^36^. Our previous study showed that the carbohydrate content of *B. odoriphaga* declines after benzothiazole treatment for 6 h^14^. In this case, DPD may play a key role in larval survival. However, the present study found that the expression of DPD was down-regulated, which suggested that the survival of *B. odoriphaga* was inhibited by benzothiazole through its effect on DPD expression.

Nucleoside diphosphate kinase (NDPK) is a ubiquitous enzyme that catalyzes the final phosphorylation of nucleoside diphosphates. This reaction uses NTP as a phosphate donor to provide sufficient nucleosides for DNA and RNA replication^38^,^39^. Moreover, NDPK is involved in several signal transduction pathways and has been described as a housekeeping enzyme that maintains a balanced pool of intracellular nucleotides^40^. The reduced level of NDPK suggested that benzothiazole disturbed the balance of nucleotide metabolic processes and interfered with the synthesis of DNA and RNA in *B. odoriphaga*.

Superoxide dismutase (SOD) is widely distributed in living organisms and catalyzes the conversion of superoxide radicals to molecular oxygen and hydrogen peroxide^41^,^42^. Thus, SOD is a critical enzyme for protecting the cell against oxygen damage (Fig. 7). The observed decrease in SOD expression implies that benzothiazole treatment might influence superoxide radical scavenging and interfere with the protection mechanism in *B. odoriphaga*. Moreover, the cytochrome P450 (CYP) enzymes play key roles in the metabolism of pharmaceutical drugs and the detoxification of xenobiotics^43^. In the present study, CYP expression was found to be decreased in the benzothiazole treatment group (Table 2), which suggested that the detoxification process is inhibited by benzothiazole. Additionally, our previous study showed that benzothiazole decreases the activity of glutathione S-transferase (GST)^14^, which also plays a critical role in detoxification pathways. Hence, the inhibition of the defense mechanism may be related to the mode of action of benzothiazole.

Cytoskeletal proteins are involved in many vital metabolic processes, such as cellular polarity, cell elongation, division, endocytosis and vesicular trafficking^44^,^45^. In our present study, the expression of two cytoskeleton-related proteins, actin-2 and calponin/transgelin, were up-regulated after exposure to benzothiazole. These results are in agreement with a number of previous studies, which demonstrated that spirotetramat, sarin, hydrogen peroxide and ethanol treatment increases actin synthesis^46^,^47^,^48^. These results indicate that actin plays an important role in the response to external stimuli and participates in innate immunity^49^. The increased expression of cytoskeleton components can reinforce the cell’s physical barrier to prevent further exposure to the stimuli and consequent injury.

The expression of 11 proteins was altered by benzothiazole treatment at both 6 and 24 h. These proteins were related to the stress response, and their possible functions are described below:

Succinyl-CoA synthetase alpha (SCS) plays a key role in the tricarboxylic acid cycle (TCA) and ketone metabolism, and SCS is the only mitochondrial enzyme that is capable of generating ATP via substrate-level phosphorylation in the absence of oxygen^50^. In the present study, the expression of SCS decreased at 6 h and increased at 24 h after exposure to benzothiazole. The metabolism of the larvae was inhibited by benzothiazole at 6 h, and after autoimmunity, the metabolic process was recovered and adapted to benzothiazole at 24 h. This finding is consistent with our motility observations (Fig. 2E and F), which indicated that the energy production of *B. odoriphaga* was decreased at 6 h and recovered at 24 h after exposure to benzothiazole.

Putative S3aE ribosomal protein (RPS3aE) is involved in many cellular processes, such as cell growth, protein synthesis and apoptosis^51^. In addition, some studies have suggested that RPS3a can alleviate copper stress in *Argopecten purpuratus*^52^. Soybean RPS3a has been associated with disease resistance and flooding tolerance^53^. A recent study suggests that RPS3aE might improve salt tolerance in three heterologous organisms^54^. In the present study, RPS3aE was up-regulated both at 6 and 24 h. Thus, RPS3aE is a multifunctional protein that plays extra-ribosomal roles in the stress response to benzothiazole.

Additionally, there were increases in proteins related to the stress response to benzothiazole. These up-regulated proteins included myosin heavy chain (MyHC), the functional myosin motor molecule, which demonstrates isoform plasticity in response to disease states^55^; putative polyadenylate-binding protein rrm superfamily (PABP), an enzyme involved in mRNA metabolism, which plays a key role in the stabilization of mRNA and promotes the initiation of translation^56^; calcium-transporting ATPase sarcoplasmic reticulum type, a membrane protein that performs the vital role of transporting Ca^2+^ up to the limiting electrochemical gradient from the cytoplasm into the sarcoplasmic reticulum^57^; putative elongation factor 1-beta2, a major translational factor and an important multifunctional protein^58^; and profilin, an actin monomer-sequestering protein that regulates actin dynamics at plasma membranes^59^. This range of changes suggested that *B. odoriphaga* mobilized the stress response mechanism to withstand ambient pressure and help it to adapt to benzothiazole.

The analysis focused on 67 proteins that exhibited different patterns of expression in response to benzothiazole only at 24 h. Many of these proteins were involved in metabolic processes, including carbohydrate metabolism, energy metabolism, amino acid metabolism, lipid metabolism and inorganic ion metabolism.

Among the proteins related to carbohydrate metabolism, many were involved in glycolysis and the pentose phosphate pathway (PPP). Putative enolase (ENO) is a key enzyme that catalyzes the ATP-generated conversion of 2-phospho-D-glycerate (2-PGA) to phosphoenolpyruvate (PEP) in the glycolytic pathway^60^. Putative transaldolase (TAL) is a nearly ubiquitous enzyme involved in the PPP, which catalyzes the transfer of a three-carbon unit (dihydroxyacetone) from donor compounds to aldehyde acceptor compounds. Furthermore, TAL also plays a crucial role in the central metabolic pathway that provides redox cofactors such as NADPH and building blocks for the biosynthesis of nucleotides and nucleic acids^61^. Pyruvate kinase (PK) is a rate-limiting enzyme that catalyzes the final step of glycolysis, which converts phosphoenolpyruvate (PEP) and ADP to ATP and pyruvate, and plays a key role in controlling glycolytic flux^62^. These proteins were up-regulated, suggesting that the surviving larvae enhanced their carbohydrate metabolism to promote the adaptation response to benzothiazole.

In total, 7 proteins involved in energy metabolism had changed levels of expression. All of these proteins underwent increased expression, including the putative IgE binding protein (epsilon BP), a galactoside-specific lectin containing a carbohydrate recognition domain^63^. Probable citrate synthase 1 (CS) is localized in the mitochondrial matrix and catalyzes the condensation of acetyl-CoA and oxaloacetate to form citrate and CoA, the first step of the Krebs cycle^64^. ATP synthase beta subunit (ATPase) catalyzes the rate-limiting step of ATP production in eukaryotic cells^65^. Malate dehydrogenase 1 (MDH) is an enzyme in the tricarboxylic acid cycle (TCA) that catalyzes the interconversion of malate (MAL) and oxaloacetic acid (OAA) using the coenzyme NAD^+^/NADH^66^. Moreover, MDH is responsible for the exchange of reducing equivalents between metabolic processes in distinct cell compartments^67^. Pyruvate carboxylase (PC) is a multifunctional, biotin-containing enzyme that catalyzes the MgATP- and bicarbonate-dependent carboxylation of pyruvate to form oxaloacetate (OAA). OAA is the key intermediate in the TCA pathway; therefore, this reaction is an important anaplerotic process in central metabolism^68^,^69^. Putative methylmalonate semialdehyde dehydrogenase (MSDH) is a mitochondrial enzyme that catalyzes the NAD-dependent oxidation of methylmalonate semialdehyde (MMSA) to propionyl-CoA through acylation and deacylation steps^70^. ATP synthase subunit beta vacuolar is a proton-translocating enzyme that plays a key role in providing energy for many secondary uptake cellular processes^30^. The up-regulation of such a wide range of energy metabolism-related proteins indicates that benzothiazole inhibits energy production in *B. odoriphaga*. Thus, more energy must be synthesized to adapt to treatment with this compound.

Many other proteins with altered expression were also identified, including those involved in amino acid metabolism, lipid metabolism and inorganic ion metabolism. These included pyrroline-5-carboxylate synthase (P5CS), a rate-limiting enzyme in proline biosynthesis, which catalyzes the coupled phosphorylation and reduction-conversion of glutamate to pyrroline-5-carboxylate (P5C)^71^. Arginine kinase (AK) is an important enzyme for maintaining energy balance and is associated with ATP regeneration, energy transport and muscle contraction in invertebrates^72^. Serine hydroxymethyltransferase (SHMT) catalyzes the interconversion of L-serine and glycine with the transfer of one-carbon units to and from tetrahydrofolate^73^. Putative 3′-phosphoadenosine 5′-phosphosulfate synthetase (PAPSS) catalyzes the synthesis of 3′-phosphoadenosine 5′-phosphosulfate (PAPS) from ATP and inorganic sulfate^74^. Na+/K+ ATPase alpha subunit is an energy-transducing ion pump^75^. Putative microtubule-associated complex regulates the dynamic structure of microtubules^76^. The enzyme 3-oxoacyl-[acyl-carrier-protein] reductase (OAR) is involved in the reductive step of fatty acid biosynthesis using NADPH as a cofactor^77^. These proteins are up-regulated, suggesting that many amino acids, lipids and inorganic ion-related enzymes enhance larval metabolism to defend against and adapt to benzothiazole treatment.

## Conclusions

In conclusion, our data revealed a comprehensive global protein response of *B. odoriphaga* upon treatment with benzothiazole. A detailed analysis of the proteins with altered expression suggested that the response of *B. odoriphaga* varies with exposure time. These proteins are divided into categories related to the action mechanism, stress mechanism and adaption mechanism. The reduction in energy metabolism, inhibition of detoxification processes and interference with DNA and RNA synthesis were potentially associated with the mode of action of benzothiazole. In addition, myosin heavy chain (MyHC), succinyl-CoA synthetase (SCS), polyA-binding protein and Ca^+^-transporting ATPase may be involved in the stress response to benzothiazole. The up-regulated expression of proteins related to carbohydrate metabolism, energy production and conversion pathways, amino acid metabolism, lipid metabolism and inorganic ion metabolism were responsible for the adaption to benzothiazole. Further studies are needed to identify the direct binding target of benzothiazole and to clearly elucidate the action mechanism of this microbial secondary metabolite.

## Materials and Methods

### Insect culture and benzothiazole treatment

A laboratory colony of *B. odoriphaga* was collected from a Chinese chive greenhouse in Liaocheng, Shandong Province, China (36°02′N, 115°30′E) in 2013. The insects were reared on fresh chive rhizomes (1 cm in length) and placed in Petri dishes, which were maintained at 25 ± 1 °C under 70 ± 5% RH and a photoperiod of 14:10 h (L:D).

Newly emerged fourth-instar larvae of *B. odoriphaga* were fumigated with the LC_30_ of benzothiazole (this was determined in our previous investigation to be 0.4729 μL/L^14^). After 6 and 24 h of continuous fumigation at 25 °C, the motility and ingestion of *B. odoriphaga* were observed and recorded for the control and benzothiazole treatment groups. In addition, surviving larvae in the benzothiazole and control treatments were collected, washed with double distilled water and stored at −80 °C. Five replications were used per benzothiazole and control treatment, and the biological replicates were performed three times.

### Protein extraction

Total proteins from each sample (n = 50 larvae/treatment, mixed from 5 replications) were pulverized thoroughly in liquid nitrogen and extracted with lysis buffer (7 M urea, 2 M thiourea, 40 mM Tris-HCl, 4% CHAPS, 1 mM PMSF, 2 mM EDTA, and 10 mM DTT; pH 8.5). The suspension was sonicated at 200 W for 15 min and then centrifuged at 30,000 × g for 15 min at 4 °C. The supernatant was transferred to a new tube, 10 mM DTT was then added, and the tube was incubated at 56 °C for 1 h. Subsequently, 55 mM iodoacetamide (IAM) was added, and the tube was incubated for 45 min in the dark. The protein was precipitated with chilled acetone for 2 h at −20 °C. After centrifugation at 30,000 × g for 20 min at 4 °C, the precipitated protein was suspended in 0.5 M tetraethylammonium bromide (TEAB) buffer. The protein concentration was determined using the Bradford dye-binding assay^78^, and 100 μg of protein was taken from each sample and digested with trypsin overnight at 37 °C before being dried under vacuum.

### iTRAQ labeling and strong cation exchange (SCX) fractionation

After trypsin digestion and desiccation, the peptides were labeled with 8-plex iTRAQ reagent (Applied Biosystems, Foster City, CA, USA) following the manufacturer’s protocol. The samples obtained from larvae challenged with distilled water for 6 h were labeled iTRAQ-113 and −115 (CON-6h1 and CON-6h2, two biological replicates). The samples obtained from larvae challenged with benzothiazole for 6 h were labeled iTRAQ-114 and −116 (BT-6h1 and BT-6h2). The samples obtained from larvae challenged with distilled water for 24 h were labeled iTRAQ-117 and −119 (CON-24h1 and CON-24h2). The samples obtained from larvae challenged with benzothiazole for 24 h were labeled iTRAQ-118 and −121 (BT-24h1 and BT-24h2). The peptides labeled with isobaric tags were incubated for 2 h at room temperature and then pooled and dried by vacuum centrifugation.

The dried peptide mixtures were dissolved in 4 mL of buffer A (25 mM NaH_2_PO_4_ in 25% ACN, pH 2.7). After centrifugation, the supernatant was loaded onto a 4.6 × 250 mm Ultremex SCX column containing 5-μm particles (Phenomenex). Elution was performed using a linear binary gradient at a flow rate of 1 mL/min with a gradient of buffer A for 10 min, 5–60% buffer B (25 mM NaH_2_PO_4_, 1 M KCl in 25% ACN, pH 2.7) for 27 min, and 60–100% buffer B for 1 min. The system was then maintained at 100% buffer B for 1 min before equilibrating with buffer A for 10 min prior to the next injection. Elution was monitored by measuring the UV absorbance at 214 nm, and fractions were collected at 1 min intervals. The eluted peptides were pooled into 20 fractions, desalted with a Strata X C18 column (Phenomenex) and dried under vacuum.

### LC-ESI-MS/MS analysis based on Triple TOF 5600

All fractions were resuspended in buffer A [0.1% formic acid (FA), 5% CAN] and centrifuged at 20,000 × g for 10 min. The average final peptide concentration was approximately 0.5 μg/μL. Then, 10 μL of supernatant was loaded onto a 2-cm C18 trap column attached to an LC-20AD nanoHPLC (Shimadzu, Kyoto, Japan) using the autosampler, and the peptides were eluted onto an analytical C18 column (inner diameter of 75 μm) that was packed in-house. The samples were loaded at 8 μL/min for 4 min, after which a 35 min gradient was run at 300 nL/min starting from 2–35% buffer B (95% ACN, 0.1% FA), followed by a 5 min linear gradient to 60%, then by a 2 min linear gradient to 80%, and maintenance in 80% buffer B for 4 min, followed by a final return to 5% buffer B in 1 min. Data acquisition was performed using a TripleTOF 5600 system (AB SCIEX, Concord, ON, Canada) fitted with a Nanospray III source (AB SCIEX) and a pulled quartz tip as the emitter (New Objectives, Woburn, MA, USA). Data were acquired using an ion-spray voltage of 2.5 kV, a curtain gas of 30 psi, a nebulizer gas of 15 psi, and an interface heater temperature of 150 °C.

### Proteomic data analysis

The raw MS/MS data were converted to mgf files and merged into a dataset using Proteome Discoverer 1.2 (Thermo Fisher Scientific, USA). Protein identification was performed using the Mascot search engine (Matrix Science, London, UK; version 2.3.02) against the NCBI Nematocera database (125804 sequences). For protein identification, a mass tolerance of 0.05 Da was permitted for intact peptide masses and 0.1 Da was permitted for fragmented ions, with allowance for one missed cleavage in the trypsin digests. Settings included Gln → pyro-Glu (N-term Q), Oxidation (M), and iTRAQ8plex (Y) as the potential variable modifications, and Carbamidomethyl (C), iTRAQ8plex (N-term), and iTRAQ8plex (K) were used as the fixed modifications. The charge states of the peptides were set to +2 and +3, and the monoisotopic mass was used. To reduce the probability of false peptide identification, only peptides at the 95% confidence interval (assessed by a Mascot probability analysis as greater than “identity”) were counted as identified. In addition, each confident protein identification involved at least one unique peptide. For protein quantitation, it was required that a protein be represented by at least two unique spectra. The quantitative protein ratios were weighted and normalized to the median ratio in Mascot. A protein was considered statistically significant only if there was a fold change of >1.20 or <0.83 in at least one biological replicate (*p* < 0.05) and if the expression trend was consistent in the rest of the biological replicates.

The identified proteins were categorized according to their Gene Ontology (GO) annotation (http://www.geneontology.org/). The Cluster of Orthologous Groups of proteins (COG) analysis was also conducted (http://www.ncbi.nlm.nih.gov/COG/). The metabolic pathway analysis of the proteins was conducted according to the Kyoto Encyclopedia of Genes and Genomes (KEGG) Pathway Database (http://www.genome.jp/kegg).

### Quantitative RT-PCR (*q*RT-PCR)

Total RNA was extracted from the frozen samples of benzothiazole-treated and control larvae using the TransZol Up Kit (Transgen, Beijing, China). For each sample, approximately 1.0 μg of total RNA was used for first-strand cDNA synthesis using the *TransScript* All-in-One First-Strand cDNA Synthesis SuperMix for *q*PCR Kit (Transgen). Gene-specific primers were designed using Primer Software Version 5.0 (Premier Biosoft International, CA, USA). The sequences of the F and R primers used are shown in Table S1. *q*RT-PCR was performed using a Bio-Rad CFX Connect Real-Time System (Bio-Rad Laboratories Inc., Hercules, CA, USA) in a total volume of 20 μL volume with 1.0 μL of cDNA, 1.0 μL of each primer, 10 μL of Tip Green *q*PCR SuperMix (Transgen) and 7.0 μL of double distilled water. The cycling conditions were 30 s at 95 °C, followed by 40 cycles of amplification (95 °C for 5 s, 58 °C for 15 s, and 72 °C for 10 s). Three technical replicates and two biological replicates were conducted for all experiments. For the normalization of gene expression, ribosomal protein S3 (RPS3) gene was used as an internal standard, and the formula 2^−∆∆Ct^ was used to determine the relative expression. The data were statistically analyzed using the Student-Newman-Keuls test (SPSS v. 13.0 for Windows) (*P* < 0.05).

## Additional Information

**How to cite this article**: Zhao, Y. *et al*. Proteomic profile of the *Bradysia odoriphaga* in response to the microbial secondary metabolite benzothiazole. *Sci. Rep*. **6**, 37730; doi: 10.1038/srep37730 (2016).

**Publisher’s note:** Springer Nature remains neutral with regard to jurisdictional claims in published maps and institutional affiliations.

## Supplementary Material

Supplementary Information

## Figures and Tables

**Table 1 t1:** Differentially expressed proteins identified by the iTRAQ analysis of *B. odoriphaga* that are related to the action mechanism of benzothiazole.

No.	Accession ID	Description	Unique Peptide	Score^a^	Coverage (%)^b^	Fold change (Mean ± SD)^c^ BT/CON^d^
	**Carbohydrate transport and metabolism**					
1	gi|563354894	triose-phosphate isomerase [*Mayetiola destructor*]	1	305	11.3	0.726 ± 0.368
	**Cytoskeleton**					
2	gi|167862461	actin-2 [*Culex quinquefasciatus*]	1	5218	38.8	1.201 ± 0.216
3	gi|197260680	calponin/transgelin [*Simulium vittatum*]	1	289	7.4	1.152 ± 0.119
	**Energy production and conversion**					
4	gi|157113604	vacuolar ATP synthase subunit H [*Aedes aegypti*]	1	140	7.4	0.765 ± 0.252
	**General function prediction only**					
5	gi|545920623	putative transporter abc superfamily [*Corethrella appendiculata*]	2	47	2.1	0.853 ± 0.117
6	gi|157110428	developmentally regulated GTP-binding protein 1 [*Aedes aegypti*]	2	117	6.3	0.694 ± 0.017
	**Inorganic ion transport and metabolism**					
7	gi|157127037	superoxide dismutase [*Aedes aegypti*]	1	94	6.5	0.807 ± 0.002
	**Lipid transport and metabolism**					
8	gi|545918281	putative peroxisomal 3-ketoacyl-CoA thiolase [*Corethrella appendiculata*]	3	183	4.4	0.869 ± 0.103
	**Nucleotide transport and metabolism**					
9	gi|157103945	dihydropyrimidine dehydrogenase [*Aedes aegypti*]	1	92	1.2	0.860 ± 0.046
10	gi|94468478	nucleoside diphosphate kinase [*Aedes aegypti*]	1	99	17.3	0.786 ± 0.020
	**Posttranslational modification, protein turnover, chaperones**					
11	gi|157131967	prohibitin [*Aedes aegypti*]	5	350	14.1	0.906 ± 0.128
	**Translation, ribosomal structure and biogenesis**					
12	gi|94468404	40 S ribosomal protein S4 [*Aedes aegypti*]	1	140	7.3	0.826 ± 0.030
	**Uncharacterized**					
13	gi|545917000	putative oligosaccharyltransferase gamma subunit [*Corethrella appendiculata*]	2	124	7.3	0.895 ± 0.100
14	gi|157361547	40 S ribosomal S30 protein-like protein [*Phlebotomus papatasi*]	2	249	8.5	0.830 ± 0.035

^a^Mascot score.

^b^Sequence coverage.

^c^Mean fold-change was calculated from two biological replicates (the same is true in in Tables 2 and 3).

^d^BT/CON: Benzothiazole VS Control.

**Table 2 t2:** Differentially expressed proteins identified by the iTRAQ analysis of *B. odoriphaga* that are related to the stress response to benzothiazole.

No.	Accession ID	Description	Unique Peptide	Score	Coverage (%)	Fold change (Mean ± SD) BT/CON	
						6 h	24 h
	**Cytoskeleton**						
15	gi|167881575	myosin heavy chain [*Culex quinquefasciatus*]	2	6019	18.3	1.291 ± 0.062	1.825 ± 0.261
	**Energy production and conversion**						
16	gi|563354922	succinyl-CoA synthetase alpha [*Mayetiola destructor*]	6	441	23.6	0.864 ± 0.180	1.439 ± 0.042
	**General function prediction only**						
17	gi|524935491	putative polyadenylate-binding protein rrm superfamily [*Anopheles aquasalis*]	2	159	8.8	1.372 ± 0.107	3.031 ± 0.029
	**Inorganic ion transport and metabolism**						
18	gi|158295513	Calcium-transporting ATPase sarcoplasmic [*Anopheles sinensis*]	2	2062	16	1.314 ± 0.035	3.379 ± 0.291
	**Posttranslational modification, protein turnover, chaperones**						
19	gi|167880127	26 S protease regulatory subunit 6 A [*Culex quinquefasciatus*]	5	239	18.2	0.698 ± 0.183	1.261 ± 0.010
20	gi|167875398	mitochondrial chaperone BCS1 [*Culex quinquefasciatus*]	1	191	2.8	1.173 ± 0.115	0.559 ± 0.000
21	gi|34867976	N-ethylmaleimide-sensitive factor [*Aedes aegypti*]	3	93	4.9	0.807 ± 0.141	0.685 ± 0.025
	**Secondary metabolites biosynthesis, transport and catabolism**						
22	gi|338841083	cytochrome P450 9J28, partial [*Aedes aegypti*]	1	55	2.1	0.830 ± 0.017	0.730 ± 0.092
	**Translation, ribosomal structure and biogenesis**						
23	gi|545921305	putative S3aE ribosomal protein [*Corethrella appendiculata*]	2	105	10.1	1.292 ± 0.144	2.662 ± 0.804
24	gi|524935628	putative elongation factor 1-beta2 [*Anopheles aquasalis*]	1	276	4	1.137 ± 0.103	1.968 ± 0.025
	**Uncharacterized**						
25	gi|157135572	profilin [*Aedes aegypti*]	2	178	15.9	1.140 ± 0.181	2.278 ± 0.827

**Table 3 t3:** Differentially expressed proteins identified by the iTRAQ analysis of *B. odoriphaga* that are related to the adaption response to benzothiazole.

No.	Accession ID	Description	Unique Peptide	Score	Coverage (%)	Fold change (Mean ± SD) BT/CON
	**Amino acid transport and metabolism**					
26	gi|54289246	pyrroline-5-carboxylate synthase, partial [*Aedes aegypti*]	2	213	4.5	1.802 ± 0.147
27	gi|284159519	arginine kinase, partial [*Coquillettidia perturbans*]	2	908	18.1	1.783 ± 0.105
28	gi|157129677	serine hydroxymethyltransferase [*Aedes aegypti*]	1	78	2.9	1.350 ± 0.112
29	gi|524934116	putative aminopeptidase [*Anopheles aquasalis*]	2	172	4.2	0.524 ± 0.052
30	gi|545916496	putative alanine aminotransferase [*Corethrella appendiculata*]	2	39	3.1	0.854 ± 0.173
	**Carbohydrate transport and metabolism**					
31	gi|157674465	putative enolase [*Lutzomyia longipalpis*]	2	1218	18.7	1.379 ± 0.096
32	gi|545917538	putative transaldolase, partial [*Corethrella appendiculata*]	1	208	6.3	1.314 ± 0.233
33	gi|563354904	pyruvate kinase [*Mayetiola destructor*]	8	928	17	1.201 ± 0.130
34	gi|405132161	glycogen phosphorylase [*Belgica antarctica*]	3	248	5.5	0.841 ± 0.066
	**Cell wall/membrane/envelope biogenesis**					
35	gi|58376929	Glucosamine–fructose-6-phosphate aminotransferase [*Anopheles gambiae*]	1	165	3.7	0.853 ± 0.146
	**Cytoskeleton**					
36	gi|31210041	Actin-related protein 3 [*Drosophila melanogaster*]	4	284	12.9	0.749 ± 0.112
	**Energy production and conversion**					
37	gi|313482947	putative IgE binding protein, partial [*Culicoides nubeculosus*]	1	318	23.8	1.569 ± 0.607
38	gi|158300600	Probable citrate synthase 1, mitochondrial [*Aedes aegypti*]	2	430	14.8	1.548 ± 0.185
39	gi|157132308	ATP synthase beta subunit [*Aedes aegypti*]	13	2473	36.9	1.529 ± 0.064
40	gi|563354932	malate dehydrogenase 1 [*Mayetiola destrauctor*]	6	200	16.9	1.519 ± 0.266
41	gi|157123846	pyruvate carboxylase [*Aedes aegypti*]	9	233	7.5	1.310 ± 0.063
42	gi|545919823	putative methylmalonate semialdehyde dehydrogenase [*Corethrella appendiculata*]	2	139	7.1	1.241 ± 0.135
43	gi|108878452	ATP synthase subunit beta vacuolar [*Aedes aegypti*]	10	570	28.6	1.231 ± 0.083
	**General function prediction only**					
44	gi|56684613	ADP ribosylation factor 79 F [*Aedes aegypti*]	4	548	31.3	1.363 ± 0.192
45	gi|545920491	putative g protein [*Corethrella appendiculata*]	5	234	13.2	1.355 ± 0.202
46	gi|157136642	ras-related protein Rab-7 [*Aedes aegypti*]	3	130	15.4	1.184 ± 0.247
47	gi|545918333	putative gtpase ran/tc4/gsp1 nuclear protein [*Corethrella appendiculata*]	5	328	28.5	1.156 ± 0.203
	**Inorganic ion transport and metabolism**					
48	gi|14906173	putative 3′-phosphoadenosine 5′-phosphosulfate synthetase, partial [*Aedes aegypti*]	1	44	4.2	1.592 ± 0.011
49	gi|157131369	Na^+^/K^+^ ATPase alpha subunit [*Aedes aegypti*]	10	619	19.8	1.505 ± 0.226
	**Intracellular trafficking, secretion, and vesicular transport**					
50	gi|545918815	putative signal peptidase i [*Corethrella appendiculata*]	3	233	18.9	0.729 ± 0.086
	**Lipid transport and metabolism**					
51	gi|524934245	putative microtubule associated complex [*Anopheles aquasalis*]	3	110	13.2	1.262 ± 0.078
52	gi|167874883	3-oxoacyl-[acyl-carrier-protein] reductase [*Culex quinquefasciatus*]	1	104	4.7	1.271 ± 0.318
	**Posttranslational modification, protein turnover, chaperones**					
53	gi|545920109	putative 60 kda heat shock protein mitochondrial [*Corethrella appendiculata*]	1	239	7.4	2.184 ± 0.233
54	gi|108868487	Small ubiquitin-related modifier 3 [*Aedes aegypti*]	1	208	12.6	1.994 ± 0.868
55	gi|157122974	prohibitin [*Aedes aegypti*]	5	144	18.4	1.976 ± 0.065
56	gi|545916790	putative atp-dependent lon protease, partial [*Corethrella appendiculata*]	5	129	5.9	1.425 ± 0.366
57	gi|2738077	heat shock protein 60 [*Culicoides variipennis*]	3	557	13.1	1.415 ± 0.105
58	gi|157106603	26 S protease regulatory subunit S10b [*Aedes aegypti*]	5	273	13.2	1.393 ± 0.136
59	gi|89212800	heat shock cognate 70 [*Rhynchosciara americana*]	13	1725	39	1.200 ± 0.040
60	gi|157131453	26 S protease regulatory subunit [*Aedes aegypti*]	1	121	14.2	0.863 ± 0.151
61	gi|108870669	Rab GDP-dissociation inhibitor [*Aedes aegypti*]	5	130	11.3	0.905 ± 0.103
	**Replication, recombination and repair**					
62	gi|167865143	ebna2 binding protein P100 [*Culex quinquefasciatus*]	2	96	2.6	0.806 ± 0.016
	**Secondary metabolites biosynthesis, transport and catabolism**					
63	gi|157115283	fatty acid synthase [*Aedes aegypti*]	2	295	1.9	1.835 ± 0.480
64	gi|584594454	cytochrome P450 6FV2 [*Chironomus kiiensis*]	1	114	4.6	0.748 ± 0.190
	**Signal transduction mechanisms**					
65	gi|58396588	14-3-3 protein epsilon [*Drosophila melanogaster*]	6	1263	22.7	0.754 ± 0.050
66	gi|94468532	myosin light chain [*Aedes aegypti*]	3	107	19.7	0.822 ± 0.023
	**Transcription**					
67	gi|58378742	Nascent polypeptide-associated complex subunit alpha [*Drosophila melanogaster*]	3	358	18	1.402 ± 0.156
	**Translation, ribosomal structure and biogenesis**					
68	gi|170030017	40 S ribosomal protein S23 [*Culex quinquefasciatus*]	2	338	15.4	1.681 ± 0.129
69	gi|568255436	hypothetical protein AND_004054 [*Anopheles darlingi*]	2	91	3.4	1.668 ± 0.091
70	gi|329669248	60 S acidic ribosomal protein P1 [*Simulium guianense*]	1	781	14.5	1.649 ± 0.204
71	gi|29839631	60 S ribosomal protein L23 [*Aedes aegypti*]	4	538	31.4	1.584 ± 0.416
72	gi|157674443	60 S acidic ribosomal protein P0-like protein [*Lutzomyia longipalpis*]	1	635	22.4	1.535 ± 0.103
73	gi|58392254	60 S ribosomal protein L12 [*Mus musculus*]	3	160	18.2	1.433 ± 0.129
74	gi|545920235	putative elongation factor 2, partial [*Corethrella appendiculata*]	2	623	12.8	1.155 ± 0.186
75	gi|56809869	ribosomal protein S9 [*Aedes albopictus*]	6	189	23.1	0.697 ± 0.145
76	gi|545920339	putative ribosomal protein l32 [*Corethrella appendiculata*]	3	436	16.4	0.739 ± 0.179
77	gi|56199506	ribosomal protein L27A, partial [*Culicoides sonorensis*]	2	398	13.2	0.777 ± 0.046
78	gi|401715292	60 s ribosomal protein L15, partial [*Nyssomyia intermedia*]	2	200	20.9	0.789 ± 0.287
79	gi|157361525	40 S ribosomal protein S15-like protein [*Phlebotomus papatasi*]	3	299	23.1	0.806 ± 0.095
80	gi|545920337	putative 60 s acidic ribosomal protein p0 [*Corethrella appendiculata*]	1	576	21.3	0.823 ± 0.240
81	gi|269146822	60 s ribosomal protein L10, partial [*Simulium nigrimanum*]	2	179	11.4	0.879 ± 0.091
	**Uncharacterized**					
82	gi|157131827	tropomyosin invertebrate [*Aedes aegypti*]	1	176	25.2	4.289 ± 2.567
83	gi|157127892	paramyosin, long form [*Aedes aegypti*]	1	146	3.9	3.158 ± 0.843
84	gi|74920601	ADP, ATP carrier protein 2 [*Anopheles gambiae*]	1	628	24.3	2.057 ± 0.155
85	gi|108884438	AAEL000311-PA [*Aedes aegypti*]	1	174	1.7	1.474 ± 0.411
86	gi|407379599	gamma-glutamylcystein synthase [*Chironomus riparius*]	1	79	2.4	1.378 ± 0.166
87	gi|167872478	mitochondrial processing peptidase beta subunit [*Culex quinquefasciatus*]	2	57	2.6	1.346 ± 0.305
88	gi|404553129	prophenoloxidase 6, partial [*Anopheles sinensis*]	2	60	7.5	0.695 ± 0.052
89	gi|157109554	titin [*Aedes aegypti*]	1	354	2.4	0.736 ± 0.095
90	gi|157117953	glutamate cysteine ligase [*Aedes aegypti*]	1	112	2.3	0.805 ± 0.250
91	gi|158295341	AGAP006103-PC [*Anopheles gambiae*]	1	202	7.5	0.850 ± 0.150
92	gi|108884446	AAEL000339-PA [*Aedes aegypti*]	1	135	2.1	0.876 ± 0.155
