# Supplementary material for: Proteomic profile of the Bradysia odoriphaga in response to the microbial secondary metabolite benzothiazole
Source: Sci Rep. 2016 Nov 24;6:37730. doi: 10.1038/srep37730 (PMC5121901; doi:10.1038/srep37730)
Supplement: Supplementary Information [file srep37730-s1.doc]

**Supplementary Information:**

Proteomic profile of the *Bradysia odoriphaga* in response to the microbial secondary metabolite benzothiazole

Yunhe Zhao ^a^, Kaidi Cui ^a^, Chunmei Xu ^a^, Qiuhong Wang ^a^, Yao Wang ^a^, Zhengqun Zhang ^b^, Feng Liu ^a^, Wei Mu ^a *^

^a^ *College of Plant Protection,* *Shandong Provincial Key Laboratory for Biology of Vegetable Diseases and Insect Pests, Shandong Agricultural University, Tai’an, Shandong 271018, P.R. China*

^b^ *College of Horticultural Science and Engineering, Shandong Agricultural University, Tai'an, Shandong 271018, PR China*

Corresponding authors:

^*^Prof. Wei Mu, College of Plant Protection, Shandong Agricultural University, 61 Daizong Street, Tai’an, Shandong 271018, P.R. China. Tel: +86-538-8242611, Email: [muwei@sdau.edu.cn](mailto:muwei@sdau.edu.cn)

**Supplementary Table S1**: Primer sequence of the target genes used for qRT-PCR analysis.

| Protein name | Primers sequence (5’ → 3’) | | Product length (bp) |
| --- | --- | --- | --- |
| TPI | F | CGCCACAACAAGCACAAG | 121 |
|  | R | GCAGTCACAGAGCCACCAT |
| V-ATPase | F | TATTGTCGCACGTCTCCAAG | 132 |
|  | R | CCCAAACATTCTCCTTACGC |
| MyHC | F | ACGGTGGCAAAGTAGGCAATT | 124 |
|  | R | GGCAAGCGTCGTAATGAAGTG |
| SCS | F | CGTAACAATGACACCCGACTT | 118 |
|  | R | TGGTTTGACGGCACCACC |
| ENO | F | GTGGGTGATGAGGGTGGATT | 106 |
|  | R | TTTCAACCTTGCCGGTGTAT |
| epsilon BP | F | TATTGTCGCACGTCTCCAAG | 132 |
|  | R | CCCAAACATTCTCCTTACGC |
| RPS3 | F | TCTACGCAGAAAAGGTGGCA | 92 |
|  | R | ACGAACGGCTAATCCACCAG |
